# **RNAPaceDB**: a dedicated database to dissect RNA velocity across diverse cell types

**DOI:** 10.1093/nar/gkaf1045

**Published:** 2025-10-21

**Authors:** Hui Liu, Honghan Zhou, Siru Yang, Weicheng Ma, Jun Wang, Nan Zhang, Huicheng Zhang, Wen Huang, Xin Zhong, Chaohan Xu, Shuyuan Wang

**Affiliations:** College of Bioinformatics Science and Technology, Harbin Medical University, Harbin 150081, China; College of Bioinformatics Science and Technology, Harbin Medical University, Harbin 150081, China; College of Bioinformatics Science and Technology, Harbin Medical University, Harbin 150081, China; College of Bioinformatics Science and Technology, Harbin Medical University, Harbin 150081, China; College of Bioinformatics Science and Technology, Harbin Medical University, Harbin 150081, China; College of Bioinformatics Science and Technology, Harbin Medical University, Harbin 150081, China; College of Bioinformatics Science and Technology, Harbin Medical University, Harbin 150081, China; College of Bioinformatics Science and Technology, Harbin Medical University, Harbin 150081, China; Department of Pathophysiology, School of Basic medicine, Harbin Medical University, Harbin 150081, China; College of Bioinformatics Science and Technology, Harbin Medical University, Harbin 150081, China; College of Bioinformatics Science and Technology, Harbin Medical University, Harbin 150081, China

## Abstract

RNA velocity, which reflects the balance between nascent RNA synthesis and mature RNA degradation, provides a kinetic perspective to explore gene expression dynamics and cellular state transitions, offering profound insights into diverse biological processes and disease mechanisms. Deciphering RNA velocity patterns across cell types and contexts will deepen our understanding of the temporal logic of gene regulation and facilitating the discovery of novel biomarkers and therapeutic targets. Here, we present RNAPaceDB (http://biocclab.cn/RNAPaceDB/), a dedicated database to dissect RNA velocity across diverse cell types. RNAPaceDB integrates single-cell RNA sequencing profiles from 144 datasets, encompassing over 2.1 million cells across 81 cell types and 41 diseases, and offers 4380 velocity stream plots derived from six computational models. Using RNAPaceDB, users can explore RNA velocity profiles of >18 000 genes across cell development processes. Additionally, RNAPaceDB provides integrated online interfaces to retrieve and analyze cell cluster distribution, cell development trajectories, cell type-specific differential velocity genes, and functional enrichments under varying conditions. Collectively, RNAPaceDB serves as a valuable resource for deciphering the temporal logic of gene expression dynamics, facilitating discoveries of regulatory mechanisms, biomarkers, and therapeutic targets in precision medicine and systems biology.

## Graphical Abstract

**Graphical Abstract:**
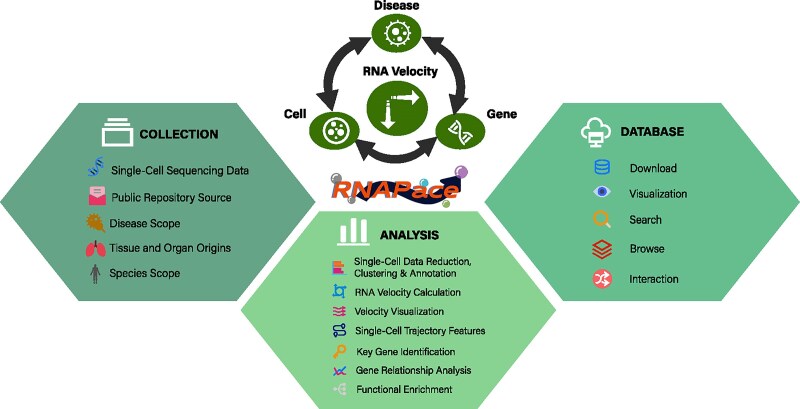

## Introduction

In recent years, single-cell RNA sequencing (scRNA-seq) has revolutionized our comprehension of gene expression with an unprecedented level of resolution. This technology allows researchers to profile the gene expression of individual cells, providing insights into cellular heterogeneity, developmental processes, and disease mechanisms [1–3]. However, while scRNA-seq provides snapshots of gene expression, it typically focuses on steady-state RNA levels, which might overlook the transient but crucial events that drive cellular state transitions and functional adaptations [4, 5]. For instance, in the context of neuronal development, certain genes are transient changes in expression during the differentiation of stem cells into neurons, making them difficult to capture in typical scRNA-seq analysis [6]. There is a growing need to develop approaches that can better capture the kinetic aspects of gene expression to gain a more complete understanding of biological systems.

RNA velocity has proposed new ways of studying cellular dynamics by measuring the balance between the synthesis of nascent RNA transcripts and the degradation of mature RNA, thereby providing a kinetic perspective on the direction and rate of transcriptional changes. This allows researchers to track gene expression dynamics in real-time, enabling prediction of cellular state transitions and trajectories. Numerous studies have demonstrated that RNA velocity offers profound insights into cellular dynamics across diverse conditions, cell types, and developmental stages [7, 8]. In oncology, RNA velocity can pinpoint cancer cells with high metastatic potential, enabling the design of precision-targeted therapies and more robust predictions of patient prognoses [9]. Alongside this, an expanding array of computational models for inferring RNA velocity—such as scVelo [9], UniTVelo [10], and DeepVelo [11]—has emerged, greatly enriching our ability to explore the kinetic basis of cell fate decisions, functional plasticity, and disease pathogenesis [12]. By bridging molecular kinetics with cellular behavior, RNA velocity reshapes our understanding of biological systems across health and disease.

Despite the immense potential of RNA velocity data, existing databases and tools primarily focus on static gene expression profiles or limited aspects of cellular trajectories [13–16]. While these resources have advanced our understanding of cellular heterogeneity and marker gene identification, they often lack the ability to uncover dynamic transcriptional trajectories and cell fate transitions. With the ongoing advancement of single-cell transcriptomics and related technologies, there is an increasing demand for a centralized platform to analyze multi-model-derived RNA velocity landscapes and their associations with cell characteristics, functional states, and disease phenotypes.

Here, we developed RNAPaceDB (http://biocclab.cn/RNAPaceDB/), a specialized database designed to dissect RNA velocity across diverse cell types and disease processes. We curated and integrated scRNA-seq profiles from publicly available datasets. By implementing unified processing pipelines and metadata standards, RNAPaceDB curated 144 datasets, encompassing over 2.1 million cells across 81 cell types and spanning 41 diseases. By providing multi-algorithmic RNA velocity estimates and integrated analytical interfaces, RNAPaceDB facilitates the investigation of gene transcriptional dynamics, cell trajectory relationships, and functional enrichment under different conditions. We anticipate that RNAPaceDB will enable a deeper understanding of gene expression dynamics and serve as a fundamental resource for the discovery of novel biomarkers and therapeutic targets in precision medicine and systems biology research.

## Materials and methods

### Data collection and processing

In RNAPaceDB, scRNA-seq expression profiles and associated metadata—including sample identifiers, organ/tissue origins, and disease types—were aggregated from Gene Expression Omnibus (GEO) [17], ArrayExpress [18], and TISCH [19]. Gene annotations were derived from GENCODE (release 41, GRCh38) [20] to identify different types of genes, including protein-coding genes, long noncoding RNAs (lncRNAs), pseudogenes, etc. RNAPaceDB utilized the t-SNE and UMAP clustering algorithms, which were implemented in the Seurat R package [21], to delineate distinct cell populations within each dataset. Cell type annotations were manually validated based on cluster-specific expression patterns of established marker genes. For RNA velocity inference, six computational models were employed to capture diverse transcriptional dynamics: scVelo_Deterministic, scVelo_Dynamical, scVelo_Stochastic, UniTVelo_Unified, UniTVelo_Independent, and DeepVelo (see Supplementary Methods for details). This multi-model implementation enables users to compare algorithms tailored to their specific biological questions. Comprehensive protocols for data collection and processing are detailed in the Supplementary Methods.

### Database construction

The web application was architected using Java Server Pages (JSP) technology running on an Apache Tomcat (v9) container, with data storage and management by a MySQL (v5.6) relational database system and SQLite (v3.50.1) for barcode-gene matrix storage. The frontend consists of a multi-page web application developed using HTML, JavaScript and CSS, with dynamic capabilities and interactive visualizations implemented through libraries including jQuery (v3.6.0), DataTables (v2.1.8), and ECharts (v5.3.3). All statistical computations and data analyses were conducted using R (v4.2.3). The platform ensures broad compatibility with contemporary web browsers, including Chrome, Edge, Firefox, and Safari, and operates without requiring user registration or login.

## Results

### Data collection and content of RNAPaceDB

**Figure 1.**
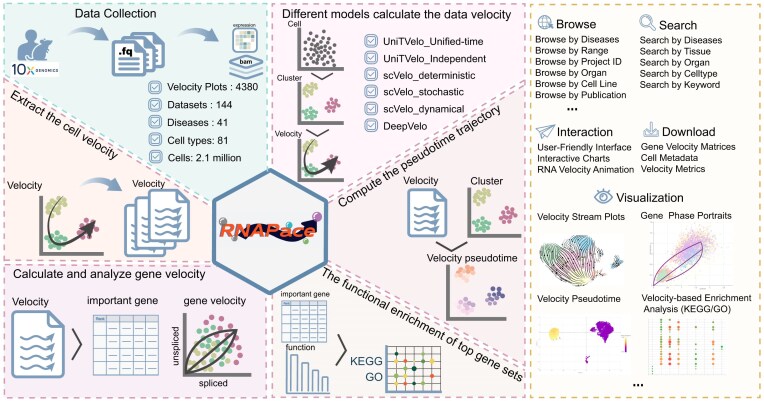
Overview of the data content and functionalities of RNAPaceDB. The left panel illustrates the data collection and content of the database. The right panel shows the navigation modules of RNAPaceDB

The design and construction of RNAPaceDB was illustrated in Fig. 1. The current version of RNAPaceDB houses 2 139 640 cells from 144 single-cell datasets spanning 41 diseases. For each dataset, the database curates clinical metadata including organ/tissue origin, sample type, and metastatic status to enable comprehensive contextual annotation. After data processing and quality control, the number of cells per dataset ranges from 1172 to 196 883 (mean, 16 425). Subsequently, RNAPaceDB conducted cell clustering and cell type annotation. Malignant cells represent the most widely represented cell type, present in 58 datasets, followed by fibroblasts (43 datasets) and T cells (38 datasets).

Next, to systematically characterize transcriptional dynamics within these cellular populations, RNAPaceDB incorporates six distinct computational models for RNA velocity inference (Fig. 1). This design allows users to explore dynamic gene expression patterns from multiple analytical perspectives, facilitating identification of velocity-driven genes, pseudotime trajectories, and cell fate transition hotspots across different cell types, disease stages, and experimental contexts. From the analytical outputs of these six models, RNAPaceDB systematically extracts and computes three core metrics to characterize transcriptional dynamics: cellular velocity values (capturing individual cells’ overall transcriptional change directionality and magnitude), gene-level velocity values (quantifying per-gene kinetic parameters like nascent-mature messenger ribonucleic acid (mRNA) balance), and pseudotime trajectories (reconstructing ordered cellular progression paths via velocity-derived constraints). These harmonized metrics across diverse models provide a multi-dimensional view of transcriptional dynamics, bridging single-gene kinetics with global cellular trajectories. In total, RNAPaceDB encompasses 4380 velocity stream plots alongside their corresponding RNA velocity profiles, covering over 18 000 genes (including protein-coding genes, lncRNAs, and pseudogenes) across 81 cell types and 41 disease contexts. RNAPaceDB further performs functional enrichment analyses on the important genes exhibiting differential velocity values between distinct clusters, thereby enabling systematic exploration of biological processes and pathways linked to dynamic transcriptional divergence. All data in RNAPaceDB are easily accessible for browsing, searching, retrieving, visualizing, and downloading (Fig. 1).

### Features and utilities of RNAPaceDB

**Figure 2.**
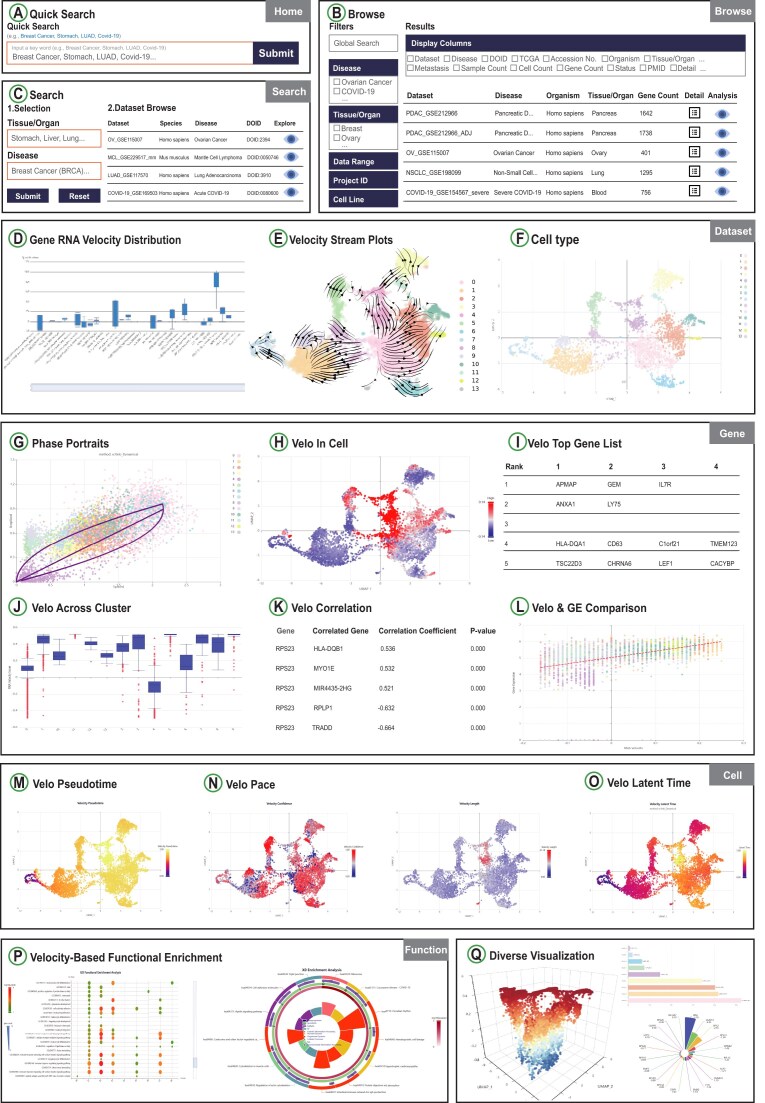
Features and utilities of RNAPaceDB. (**A**) Quick Search entry for datasets on the home page. (**B**) Filter-based browse for datasets in the “Data Browser” page. (**C**) Detailed search interface for precise dataset querying on the “Velocity Analysis” page. (**D–** **Q**) A panel of user-friendly visualization modules in RNAPaceDB.

To ensure rapid and flexible data retrieval, RNAPaceDB offers three user-friendly access strategies: “Quick Search” (via the Home page search box) for keyword-based dataset retrieval using terms like disease types or tissues (Fig. 2A); “Data Browser” interface with predefined modules (Disease, Tissue/Organ, etc.) to refine selections by dataset attributes, with each dataset accompanied by detailed description (Fig. 2B); and the “Velocity Analysis” interface, which retrieves analysis results by diseases, tissues, or organs and displays real-time RNA velocity visualizations (Fig. 2C). RNAPaceDB supports cross-dataset, gene-specific RNA velocity comparison. Users can initiate this analysis by inputting an interesting gene (e.g. MYC) or selecting one from the built-in gene list via either the home page’s “Quick Search” box or the dedicated “Velocity Analysis” page. The system then automatically retrieves and visualizes the RNA velocity distribution of this gene across all relevant datasets (Fig. 2D). The “Velocity Analysis” interface offer four modules: Dataset, Gene, Cell, and Function. The Dataset module focuses on the holistic RNA velocity landscape inferred by the six computational models for a selected dataset, equipped with two sub-modules: “Velocity Stream Plots” and “Cell Type.” The “Velocity Stream Plots” section displays velocity stream distributions across cells, annotated with cell clusters and cell types (Fig. 2E). The “Cell Type” section incorporates cell clusters and annotations, along with statistical summaries of cell counts and proportions for each cluster or cell type to support the quantitative assessment of cellular composition (Fig. 2F). The Gene module enables in-depth analysis of RNA velocity characteristics for important genes, equipped with six sub-modules: “Phase Portraits,” “Velo In Cell,” “Velo Top Gene List,” “Velo Across Cluster,” “Velo Correlation,” and “Velo & GE Comparison.” Important genes are defined as the union of cluster-specific differential velocity genes, where these genes are identified as the top 100 ranked candidates from differential velocity *t*-tests performed for each individual cluster. The “Phase Portraits” section illustrates the relationship between unspliced (nascent) and spliced (mature) mRNA level of the selected gene (Fig. 2G). The “Velo in Cell” section displays the RNA velocity profile of genes inferred by the six computational models for each cell, accompanied by gene expression data for comparison (Fig. 2H). The “Velo Top Gene list” presents the top 100 ranked genes for each cluster, derived from differential velocity *t*-tests (Fig. 2I). The “Velo Across Cluster” displays the RNA velocity of a selected gene across different cell types or clusters (Fig. 2J). The “Velo Correlation” section presents the RNA velocity correlation between a selected gene and other important genes (Fig. 2K). The “Velo & GE Comparison” exhibits the correlation between RNA velocity and gene expression for a selected gene (measured by Pearson Correlation Coefficient) (Fig. 2L). The Cell module presents the dynamic characteristics of single cells derived from RNA velocity, equipped with four sub-modules: “Velo Pseudotime,” “Velo Pace,” and “Velo Latent Time.” The “Velo Pseudotime” sub-module reconstructs cellular progression paths based on velocity constraints, reflecting the inferred order of cell state transitions (Fig. 2M). The “Velo Pace” sub-module provides the differentiation speed or rate of individual cells, comprising two sections: “Velo Length” and “Velo Confidence.” The “Velo Length” section reflects the speed at which cells differentiate, where lower values indicate a slower differentiation pace and higher values indicate a faster pace. The “Velo Confidence” section indicates whether the direction is determined or undetermined, with higher values corresponding to greater confidence (Fig. 2N). The “Velo Latent Time” sub-module, computed via scVelo’s Dynamical module, recovers the latent time of underlying cellular processes, where a lower value indicates earlier differentiation (Fig. 2O). The Function module performs KEGG pathway [22] and GO term [23] enrichment analyses using important genes with differential RNA velocity, linking dynamic transcriptional patterns to biological processes, molecular functions, and cellular components to facilitate mechanistic interpretation (Fig. 2P). Notably, users can interactively explore single-cell details by clicking on individual cells in the visualization panels to examine cell-specific RNA velocity metrics and transcriptional characteristics (Fig. 2Q). In addition, RNAPaceDB incorporates diverse visualization formats, including scatter plots, stream plots, 3D interactive visualizations, correlation matrices, to comprehensively present RNA velocity data, cell clusters, gene expression profiles, and functional enrichments. These visualization tools not only enhance intuitive understanding of complex transcriptional dynamics but also enable both qualitative observation and quantitative analysis of RNA velocity-driven cellular processes. Together, these advanced features establish RNAPaceDB as a comprehensive platform for investigating RNA velocity across diverse cell types and disease contexts-spanning from dataset retrieval and exploratory analysis to in-depth functional interpretation at single-cell resolution.

### Example application

**Figure 3.**
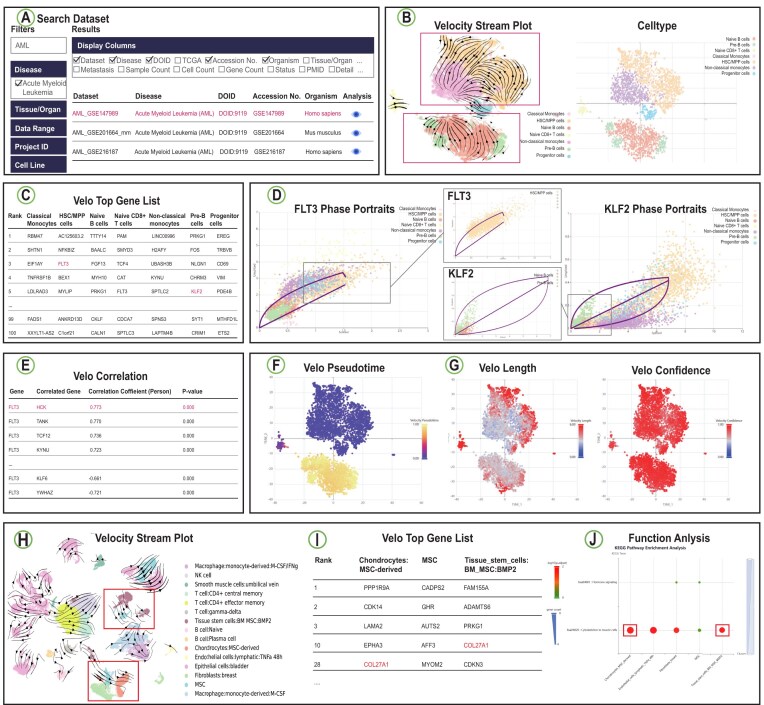
Example application of RNAPaceDB. (**A**) Exploration of AML dataset. (**B**) Velocity Stream Plots of the AML_GSE147989 dataset, generated using the DeepVelo model, scType cell type annotation method, and t-SNE clustering algorithm. (**C**) the Velo Top Gene List (selected examples) for the AML_GSE147989 dataset. (**D**) The gene FLT3 and KLF2 phase portraits. (**E**) Velocity correlation matrix (selected examples) for gene FLT3. (**F**) RNA velocity-constrained pseudotime analysis for AML_GSE147989 dataset. (**G**) Velo Pace analysis for AML_GSE147989 dataset. (**H**) Velocity Stream Plots of the TNBC_GSE148673 dataset, generated using the scVelo_Stochastic model, SingleR cell type annotation method, and t-SNE clustering algorithm. (**I**) the Velo Top Gene List (selected examples) for TNBC_GSE148673 dataset. (**J**) Function enrichment (selected examples) for TNBC_GSE148673 dataset.

To demonstrate the potential application of RNAPaceDB in dissecting RNA velocity dynamics and exploring transcriptional trajectories, we performed an analysis on a scRNA-seq dataset of Acute Myeloid Leukemia (AML). AML is an aggressive hematological malignancy characterized by clonal expansion of immature myeloid blasts disrupting normal hematopoiesis, with high incidence in adult leukemias and variable prognosis, highlighting the need for insights into its pathogenesis to develop targeted therapies [24]. We browse the database by selecting “Acute Myeloid Leukemia” from the Disease menu on the “Data Browser” page, then choosing the dataset AML_GSE147989 (Fig. 3A). On the Dataset module of the “Velocity Analysis” page, we selected the DeepVelo model, scType cell type annotation method, and t-SNE clustering algorithm. The resulting “Velocity Stream Plots” revealed definitive cellular kinetics: hematopoietic stem/progenitor cells (HSC/MPP) exhibited pronounced velocity vectors directed toward nonclassical monocytes, while Pre-B cells demonstrated targeted progression to naive B cells. This spatial patterning aligns precisely with established hematopoietic differentiation hierarchies—HSC/MPP inherently possess multilineage differentiation capacity with strong myeloid (including monocytic) bias, and Pre-B cells represent a transitional stage preceding immunocompetent naive B lymphocytes (Fig. 3B). Based on these observations, we hypothesized that active differentiation trajectories exist from HSC/MPP to nonclassical monocytes and from Pre-B to naive B cells within the AML microenvironment, prompting rigorous validation through integrated analytical modules. We utilized the “Velo Top Gene List” sub-module within the gene module to extract the top 100 cluster-specific differential velocity genes for HSC/MPP, nonclassical monocytes, Pre-B cells, and naive B cells. We observed that FLT3 was prioritized in HSC/MPP cells; notably, FLT3 ligand binding is reported to activate PI3K-Akt, RAS-MAPK, and STAT3/STAT5 signaling cascades, directly promoting monocytic commitment from progenitors [25] (Fig. 3C). Similarly, KLF2 is notable as it orchestrates B-cell receptor (BCR)-dependent maturation—potentiating signaling via CD21/CD22 coreceptors, with loss-of-function studies confirming maturation arrest at the Pre-B stage [26] (Fig. 3C). Phase portraits analysis revealed dynamic transcriptional states: FLT3 exhibited high transcriptional induction in HSC/MPP, while KLF2 transitioned from active transcription in Pre-B cells to silenced states in naive B cells (Fig. 3D). Velocity correlation analysis also revealed significant synergistic regulatory pairs: FLT3-HCK (*r* = 0.773, *P*< 1e-15). It has been reported that FLT3 phosphorylates HCK to activate monocytic maturation programs, where HCK functions as a nonredundant effector downstream of oncogenic FLT3 signaling [27] (Fig. 3E). RNA velocity-constrained pseudotime analysis reconstructed continuous differentiation trajectories: HSC/MPP→nonclassical monocytes, with pseudotime values increasing monotonically from HSC/MPP through transitional states to nonclassical monocytes. Similarly, pseudotime progression from pre-B to naive B cells further confirmed the developmental ordering of these lineages (Fig. 3F). Velo Pace analysis revealed kinetic signatures wherein maximum differentiation velocity occurred in transitional cell states, while directional uncertainty peaked at bifurcation points—consistent with lineage commitment events (Fig. 3G). We additionally conducted an analysis on TNBC_GSE148673—a dataset of triple-negative breast cancer (TNBC). We selected the scVelo_Stochastic model, SingleR cell type annotation method, and t-SNE clustering algorithm. The “Velocity Stream Plots” revealed directional transitions of mesenchymal stem cell (MSC) toward two distinct cell fates: one trajectory toward Tissue_stem_cells: BM_MSC:BMP2 (bone marrow-derived MSCs responsive to BMP2, a key osteogenic differentiation factor) and a second toward Chondrocytes: MSC-derived (chondrocyte-like cells differentiated from MSCs) (Fig. 3H). This observation is biologically meaningful in the context of TNBC: MSCs in the tumor microenvironment (TME) are known to undergo lineage plasticity, differentiating into osteoblasts, chondrocytes, or adipocytes to remodel the extracellular matrix (ECM)—a process that promotes tumor invasion and chemoresistance [28]. The “Velo Top Gene List” sub-module exhibited COL27A1 as a top-ranked velocity-driving gene in both target cell populations, including both Tissue_stem_cells:BM_MSC:BMP2 and Chondrocytes:MSC-derived (Fig. 3I). This observation aligns with the well-established biological role of COL27A1: as a core driver of chondrocyte differentiation and ECM maturation, COL27A1 not only regulates collagen fibril assembly in developing cartilage but also serves as a key mediator of MSC commitment to the chondrocyte lineage [29]. Its high velocity contribution confirms it as a key regulator of the observed MSC transitions. Functional enrichment analysis of important genes in Tissue_stem_cells:BM_MSC:BMP2 identified significant enrichment of the hsa04820: Cytoskeleton in muscle cells pathway (Fig. 3J). This pathway is indirectly linked to MSC osteogenic differentiation: cytoskeletal remodeling (e.g. actin filament reorganization) is a prerequisite for MSC morphological changes during osteoblast differentiation, and activation of this pathway promotes the maturation of osteoblast precursors into functional osteoblasts. This enrichment further validates the biological relevance of the BMP2-responsive MSC trajectory, as BMP2 is a canonical inducer of osteogenic differentiation in MSCs. Together, these two case studies—spanning immune cell activation and TME remodeling—exemplify RNAPaceDB’s capacity to support research across diverse biological contexts. This breadth of application not only demonstrates the database’s versatility but also validates its utility as a powerful resource for dissecting complex cellular dynamics in disease-relevant systems.

## Discussion

ScRNA-seq has revolutionized our ability to dissect cellular heterogeneity and gene expression patterns in complex biological systems, from normal development to disease pathogenesis. However, capturing the dynamic nature of transcriptional regulation—such as the transient gene expression changes that drive cell fate transitions—remains a key challenge, as traditional scRNA-seq primarily provides static snapshots of steady-state RNA levels. RNA velocity, by quantifying the balance between nascent RNA synthesis and mature RNA degradation, offers a powerful solution to this limitation, enabling the inference of transcriptional dynamics and cellular trajectory directionality. Despite its potential, the lack of centralized resources integrating RNA velocity data across diverse cell types and diseases has hindered systematic exploration of its biological implications.

To address this gap, we developed RNAPaceDB, a dedicated database that consolidates large-scale scRNA-seq data and multi-algorithmic RNA velocity profiles. Unlike existing databases that focus on static gene expression or limited trajectory analyses, RNAPaceDB provided 4380 velocity stream plots, alongside their corresponding RNA velocity profiles covering over 18 000 genes. By incorporating six computational models, RNAPaceDB enables users to access velocity estimates derived from different algorithmic assumptions, enhancing the robustness of dynamic transcriptional inference. Users can directly choose a model based on the biological scenario of their research (e.g. selecting UniTVelo_Independent for cell cycle studies, where gene-specific dynamics are prominent) and reference the model's documented suitability in the Supplementary Materials. For scenarios with unclear biological characteristics (e.g. novel cell populations with uncharacterized transcriptional dynamics), users can access results from all six models, download the raw velocity profile, and prioritize conclusions supported by consistent results across the majority of models. Notably, due to the distinct underlying assumptions of each model, contradictory velocity results may occasionally emerge when analyzing the same cell population. Rather than forcing artificial integration or reconciliation of these discrepancies (which could obscure model-specific biases and distort biological interpretations), RNAPaceDB presents all model outputs in parallel. Each output is paired with detailed documentation of the model’s core assumptions (e.g. latent time framework and steady-state requirements) in the Help page, ensuring transparency. This design allows users to compare conflicting results within the context of model characteristics, interpret why discrepancies arise (e.g. a steady-state model vs. a nonstationary model yielding divergent trends), and make informed decisions—such as leaning on consensus results across models when addressing well-defined biological questions, or prioritizing a model whose assumptions match their system’s known dynamics. The interactive tools enable users to explore cell cluster distributions, developmental trajectories, cell type-specific differential velocity genes, and functional enrichments under varying conditions. Importantly, RNAPaceDB is designed to serve diverse researchers: cancer biologists can dissect velocity dynamics of oncogenes during tumor progression, immunologists can trace immune cell differentiation trajectories, and computational biologists can benchmark new velocity algorithms using its standardized data. This tailored utility ensures the database meets the distinct needs of these core user groups, while also lowering the barrier for noncomputational researchers to explore transcriptional dynamics.

It should also be pointed out that RNAPaceDB’s reliance on public datasets may introduce a potential limitation: an inherent bias toward well-studied diseases and cell types. This bias may lead to uneven representation of these biological categories, which in turn could influence downstream RNA velocity analyses. RNAPaceDB will be proactively updated quarterly to incorporate newly published high-quality single-cell datasets, with a specific focus on supplementing underrepresented diseases (e.g. rare cancers and nonmalignant conditions) and cell types, aiming to gradually balance the distribution of diseases and cell types across the database. Recognizing the significant value of model organisms for exploring the evolutionary conservation of transcriptional regulation, we plan to systematically integrate single-cell datasets from additional widely used model organisms—including zebrafish (a pivotal model for developmental biology) and Drosophila (a key system for dissecting conserved regulatory networks)—in future quarterly updates. Additionally, while the current platform integrates six velocity models, it will expand to include emerging algorithms such as cell2fate [30], cellDancer [31], VeloVI [32], TIVelo [33], and SymVelo [34]—further enhancing the depth and versatility of dynamic inference. Furthermore, as spatial transcriptomics technologies (e.g. 10× Visium, Slide-seq) that enabling precise mapping of gene expression to specific tissue locations advance rapidly, innovative models for jointly inferring spatial and temporal dynamics of cell fate transitions from spatial transcriptomic data have emerged, such as Topological Velocity Inference (TopoVelo) [35], spVelo [36], and SIRV [37]. While the current version of RNAPaceDB focuses primarily on RNA velocity profiles derived from single-cell RNA-seq and does not yet support spatial RNA velocity analysis, we have prioritized this functionality in our long-term development roadmap, forging critical connections between transcriptional dynamics and tissue architecture. In summary, RNAPaceDB provides a centralized, multi-model platform for exploring RNA velocity across diverse biological contexts, empowering researchers to dissect temporal gene regulatory mechanisms and identify novel biomarkers and therapeutic targets. We anticipate that RNAPaceDB will become an essential resource for the systems biology and precision medicine communities, driving discoveries in dynamic cellular processes.

## Supplementary Material

gkaf1045_Supplemental_File

## Data Availability

RNAPaceDB is freely available online at http://biocclab.cn/RNAPaceDB/. This website is free and open to all users and there is no login requirement.

## Supplementary data

Supplementary data is available at NAR online.

## References

[1] Jovic D, Liang X, Zeng H et al. Single-cell RNA sequencing technologies and applications: a brief overview. Clin Transl Med. 2022; 12:e694 10.1002/ctm2.694. 35352511 PMC8964935

[2] Tirosh I, Suva ML Cancer cell states: lessons from ten years of single-cell RNA-sequencing of human tumors. Cancer Cell. 2024; 42:1497–506. 10.1016/j.ccell.2024.08.005. 39214095

[3] Gulati GS, D'Silva JP, Liu Y et al. Profiling cell identity and tissue architecture with single-cell and spatial transcriptomics. Nat Rev Mol Cell Biol. 2025; 26:11–31. 10.1038/s41580-024-00768-2. 39169166

[4] La Manno G, Soldatov R, Zeisel A et al. RNA velocity of single cells. Nature. 2018; 560:494–8. 10.1038/s41586-018-0414-6. 30089906 PMC6130801

[5] Kharchenko PV The triumphs and limitations of computational methods for scRNA-seq. Nat Methods. 2021; 18:723–32. 10.1038/s41592-021-01171-x. 34155396

[6] Zywitza V, Misios A, Bunatyan L et al. Single-cell transcriptomics characterizes cell types in the subventricular zone and uncovers molecular defects impairing adult neurogenesis. Cell Rep. 2018; 25:2457–69. 10.1016/j.celrep.2018.11.003. 30485812

[7] Ghersi JJ, Baldissera G, Hintzen J et al. Haematopoietic stem and progenitor cell heterogeneity is inherited from the embryonic endothelium. Nat Cell Biol. 2023; 25:1135–45. 10.1038/s41556-023-01187-9. 37460694 PMC10415179

[8] Eze UC, Bhaduri A, Haeussler M et al. Single-cell atlas of early human brain development highlights heterogeneity of human neuroepithelial cells and early radial glia. Nat Neurosci. 2021; 24:584–94. 10.1038/s41593-020-00794-1. 33723434 PMC8012207

[9] Bergen V, Lange M, Peidli S et al. Generalizing RNA velocity to transient cell states through dynamical modeling. Nat Biotechnol. 2020; 38:1408–14. 10.1038/s41587-020-0591-3. 32747759

[10] Gao M, Qiao C, Huang Y UniTVelo: temporally unified RNA velocity reinforces single-cell trajectory inference. Nat Commun. 2022; 13:6586 10.1038/s41467-022-34188-7. 36329018 PMC9633790

[11] Cui H, Maan H, Vladoiu MC et al. DeepVelo: deep learning extends RNA velocity to multi-lineage systems with cell-specific kinetics. Genome Biol. 2024; 25:27 10.1186/s13059-023-03148-9. 38243313 PMC10799431

[12] Wang Y, Li J, Zha H et al. Paradigms, innovations, and biological applications of RNA velocity: a comprehensive review. Brief Bioinform. 2025; 26:bbaf339 10.1093/bib/bbaf339. 40668554 PMC12265890

[13] Li M, Zhang X, Ang KS et al. DISCO: a database of deeply integrated human single-cell omics data. Nucleic Acids Res. 2022; 50:D596–602. 10.1093/nar/gkab1020. 34791375 PMC8728243

[14] Zeng J, Zhang Y, Shang Y et al. CancerSCEM: a database of single-cell expression map across various human cancers. Nucleic Acids Res. 2022; 50:D1147–55. 10.1093/nar/gkab905. 34643725 PMC8728207

[15] Guo Q, Wang P, Liu Q et al. CellTracer: a comprehensive database to dissect the causative multilevel interplay contributing to cell development trajectories. Nucleic Acids Res. 2023; 51:D861–9. 10.1093/nar/gkac892. 36243976 PMC9825461

[16] Hu C, Li T, Xu Y et al. CellMarker 2.0: an updated database of manually curated cell markers in human/mouse and web tools based on scRNA-seq data. Nucleic Acids Res. 2023; 51:D870–6. 10.1093/nar/gkac947. 36300619 PMC9825416

[17] Clough E, Barrett T, Wilhite SE et al. NCBI GEO: archive for gene expression and epigenomics data sets: 23-year update. Nucleic Acids Res. 2024; 52:D138–44. 10.1093/nar/gkad965. 37933855 PMC10767856

[18] Athar A, Fullgrabe A, George N et al. ArrayExpress update—from bulk to single-cell expression data. Nucleic Acids Res. 2019; 47:D711–5. 10.1093/nar/gky964. 30357387 PMC6323929

[19] Sun D, Wang J, Han Y et al. TISCH: a comprehensive web resource enabling interactive single-cell transcriptome visualization of tumor microenvironment. Nucleic Acids Res. 2021; 49:D1420–30. 10.1093/nar/gkaa1020. 33179754 PMC7778907

[20] Mudge JM, Carbonell-Sala S, Diekhans M et al. GENCODE 2025: reference gene annotation for human and mouse. Nucleic Acids Res. 2025; 53:D966–75. 10.1093/nar/gkae1078. 39565199 PMC11701607

[21] Butler A, Hoffman P, Smibert P et al. Integrating single-cell transcriptomic data across different conditions, technologies, and species. Nat Biotechnol. 2018; 36:411–20. 10.1038/nbt.4096. 29608179 PMC6700744

[22] Kanehisa M, Furumichi M, Tanabe M et al. KEGG: new perspectives on genomes, pathways, diseases and drugs. Nucleic Acids Res. 2017; 45:D353–61. 10.1093/nar/gkw1092. 27899662 PMC5210567

[23] Ashburner M, Ball CA, Blake JA et al. Gene ontology: tool for the unification of biology. The Gene Ontology Consortium. Nat Genet. 2000; 25:25–9. 10.1038/75556. 10802651 PMC3037419

[24] Newell LF, Cook RJ Advances in acute myeloid leukemia. BMJ. 2021; 375:n2026. 34615640 10.1136/bmj.n2026

[25] Momenilandi M, Levy R, Sobrino S et al. FLT3L governs the development of partially overlapping hematopoietic lineages in humans and mice. Cell. 2024; 187:2817–37. 10.1016/j.cell.2024.04.009. 38701783 PMC11149630

[26] Winkelmann R, Sandrock L, Kirberg J et al. KLF2–a negative regulator of pre-B cell clonal expansion and B cell activation. PLoS One. 2014; 9:e97953 10.1371/journal.pone.0097953. 24874925 PMC4038547

[27] Lopez S, Voisset E, Tisserand JC et al. An essential pathway links FLT3-ITD, HCK and CDK6 in acute myeloid leukemia. Oncotarget. 2016; 7:51163–73. 10.18632/oncotarget.9965. 27323399 PMC5239466

[28] Noth U, Osyczka AM, Tuli R et al. Multilineage mesenchymal differentiation potential of human trabecular bone-derived cells. J Orthopaedic Res. 2002; 20:1060–9. 10.1016/S0736-0266(02)00018-9. 12382974

[29] Hjorten R, Hansen U, Underwood RA et al. Type XXVII collagen at the transition of cartilage to bone during skeletogenesis. Bone. 2007; 41:535–42. 10.1016/j.bone.2007.06.024. 17693149 PMC2030487

[30] Aivazidis A, Memi F, Kleshchevnikov V et al. Cell2fate infers RNA velocity modules to improve cell fate prediction. Nat Methods. 2025; 22:698–707. 10.1038/s41592-025-02608-3. 40032996 PMC11978503

[31] Li S, Zhang P, Chen W et al. A relay velocity model infers cell-dependent RNA velocity. Nat Biotechnol. 2024; 42:99–108. 10.1038/s41587-023-01728-5. 37012448 PMC10545816

[32] Gayoso A, Weiler P, Lotfollahi M et al. Deep generative modeling of transcriptional dynamics for RNA velocity analysis in single cells. Nat Methods. 2024; 21:50–9. 10.1038/s41592-023-01994-w. 37735568 PMC10776389

[33] Ge M, Miao J, Qi J et al. TIVelo: RNA velocity estimation leveraging cluster-level trajectory inference. Nat Commun. 2025; 16:6258 10.1038/s41467-025-61628-x. 40624054 PMC12234748

[34] Xie C, Yang Y, Yu H et al. RNA velocity prediction via neural ordinary differential equation. iScience. 2024; 27:109635 10.1016/j.isci.2024.109635. 38623336 PMC11016905

[35] Gu Y, Liu J, Lee KH et al. Topological velocity inference from spatial transcriptomic data. Nat Biotechnol. 2025; 10.1038/s41587-025-02688-8. 40670711

[36] Long W, Liu T, Xue L et al. spVelo: RNA velocity inference for multi-batch spatial transcriptomics data. Genome Biol. 2025; 26:239 10.1186/s13059-025-03701-8. 40790237 PMC12337411

[37] Abdelaal T, Grossouw LM, Pasterkamp RJ et al. SIRV: spatial inference of RNA velocity at the single-cell resolution. NAR Genom Bioinform. 2024; 6:lqae100 10.1093/nargab/lqae100. 39108639 PMC11302586

