# Supplementary material for: RNAPaceDB: a dedicated database to dissect RNA velocity across diverse cell types
Source: Nucleic Acids Res. 2025 Oct 21;54(D1):D1242–50. doi: 10.1093/nar/gkaf1045 (PMC12807595; doi:10.1093/nar/gkaf1045)
Supplement: gkaf1045_Supplemental_File [file gkaf1045_supplemental_file.pdf]

Supplementary Methods

Data Collection and Preprocessing

We systematically collected raw single-cell RNA sequencing (scRNA-seq) datasets through the Gene Expression Omnibus (GEO, https://www.ncbi.nlm.nih.govgeo/), European Nucleotide Archive (ENA, https://www.ebi.ac.uk/ena/browser/home), Sequence Read Archive (SRA, https://www.ncbi.nlm.nih.gov/sra), and published studies. Following initial collection, we applied strict curation criteria to ensure dataset quality and suitability for RNA velocity analysis: first, regarding data completeness, we only retained datasets that provide raw count matrices (or normalized expression matrices) alongside detailed cell metadata—including cell type annotations, sample conditions, and disease status—as these components are essential for both accurate RNA velocity calculation and meaningful downstream interpretation of results; second, to meet the technical demands of RNA velocity inference (which relies on capturing subtle differences between nascent and mature RNA signals), we prioritized datasets with a minimum of 50,000 reads per cell and detection of ⩾1,000 genes per cell, thresholds that ensure sufficient sequencing coverage for reliable velocity estimation. For each dataset, relevant metadata—including sample ID, organ/tissue origin, primary/metastatic site, and cell type—were integrated into RNAPaceDB. Post-curation, all datasets underwent a standardized processing pipeline: 1) Raw scRNA-seq data were processed using Cell Ranger software (10x Genomics, v6.1.2) for sequence alignment (against the GRCh38 reference genome) and gene expression quantification, generating raw UMI count matrices. This was followed by quality control (QC) using the R package Seurat (v4.3.0) to filter low-quality cells, with those exhibiting >20% mitochondrial gene expression or <200 detected genes excluded to remove apoptotic cells or technical artifacts; 2) For each dataset, Seurat's 'NormalizeData' function with the "LogNormalize" method (scaling factor = 10,000) was applied to normalize raw UMI counts, eliminating differences in sequencing depth across cells while preserving relative expression patterns; 3) Genes were sorted by the coefficient of variation (CV) of their normalized expression across cells, with the top 3,000 most variable genes retained to focus on biologically informative signals and reduce computational noise from low-variability genes; 4) To address technical variability across experimental batches, sequencing platforms, or laboratories, batch effect correction was performed using the R package Harmony (v1.2.0): principal component analysis (PCA) was first conducted on the 3,000 highly variable genes

(retaining the top 50 PCA dimensions), followed by Harmony's iterative probabilistic model to align cellular distributions across batches; 5) For downstream visualization and velocity calculation, batch-corrected PCA dimensions (top 30) were used to perform UMAP via Seurat's 'RunUMAP' function (with parameters 'n.neighbors = 30' and 'min.dist = 0.3'), generating standardized low-dimensional embeddings that also served as input for RNA velocity models (e.g., Velocyto, scVelo) to infer trajectory directionality. After raw data QC and normalization, RNAPaceDB documented 144 datasets, encompassing over 2.1 million cells across 81 cell types and spanning 41 diseases.

Cell Clustering Analysis

Unsupervised dimensionality reduction and clustering of gene expression profiles were performed using the R package Seurat (v4.0.2; https://satijalab.org/seurat/). The workflow comprised the following steps: Principal Component Analysis (PCA)—optimal principal components (PCs) were identified using the JackStraw and ElbowPlot functions from Seurat. Clustering — cell clusters were generated via the FindClusters algorithm with a resolution parameter set to 0.1. Visualization — dimensionality reduction results were visualized using t-distributed Stochastic Neighbor Embedding (t-SNE) and Uniform Manifold Approximation and Projection (UMAP) implemented through the RunTSNE and RunUMAP functions, respectively.

Cell Type Annotation

Cell type annotation in RNAPace utilizes two complementary approaches:

(i) SingleR, an automated cell type labeling tool for scRNA-seq data, which leverages reference-based annotation by mapping the transcriptomic profiles of query cells to well-curated, established transcriptomic datasets. These reference datasets typically include thoroughly characterized cell populations with known identities, allowing SingleR to assign cell types based on transcriptional similarity, thereby ensuring annotations align with previously validated biological classifications.

(ii) Sctype (Single Cell Type Identifier), which implements a marker gene enrichment-based strategy for automated annotation. This method quantifies the enrichment level of predefined cell type-specific marker gene sets within the transcriptional profiles of target cells, generating a score that reflects the consistency between the query cells and each reference cell type. By prioritizing cell types with the highest enrichment scores, Sctype enables rapid, standardized classification.

RNA Velocity Analysis

(i) Data Preprocessing

All datasets were processed using the standard scVelo pipeline, which consisted of the following steps: First, size normalization was applied to the unspliced and spliced count matrices across all cells using the scv.pp.normalize function. Next, high-quality genes were selected based on a threshold requiring detectable expression (with both spliced and unspliced counts) in at least 30 cells. A K-nearest neighbor (KNN) graph (default: K=30 neighbors) was then constructed using Euclidean distances derived from principal component analysis (PCA; default: 30 principal components), which was applied to the logarithmically transformed spliced mRNA count matrix. To mitigate the high noise inherent in scRNA-seq data, raw counts were smoothed prior to velocity estimation to stabilize variance. This smoothing was implemented by calculating the first moment for each cell via the KNN graph, where the spliced and unspliced RNA values of each cell were replaced by the average values of its neighboring cells. These preprocessing steps were executed using the scv.pp.filter_and_normalize() and scv.pp.moments() functions from the scVelo package.

(ii) RNA Velocity inference

We utilized six distinct computational models, implemented across three computational methods, to derive RNA velocity profiles: scVelo_Deterministic, scVelo_Dynamical, scVelo_Stochastic, UniTVelo_Unified, UniTVelo_Independent, and DeepVelo.

scVelo infers velocity by distinguishing unspliced and spliced mRNA dynamics, with three models optimized for varying assumptions about transcriptional “steady-state” vs. “dynamic” regimes:

scVelo_Deterministic: This model designed to better capture steady states by treating transcription/splicing/degradation as probabilistic events, approximating the resulting Markov process via moment equations that incorporate second-order moments to leverage both unspliced-spliced mRNA balance and their covariation. It is suitable for biological scenarios where the system is relatively close to a steady state, such as the study of certain adult tissues with relatively stable cell populations. For example, in the research of normal liver tissue cell populations, where the transcriptional and splicing processes of cells are in a relatively stable state with minimal fluctuations, the scVelo_Deterministic model analyzes RNA velocity to more

accurately reflect gene kinetic changes in this stable state.

scVelo_Dynamical: This model efficiently estimates RNA velocity by quantifying how the observed unspliced-spliced mRNA ratio deviates from steady-state equilibrium. It operates under the key assumption that transcriptional phases are sufficiently long for cells to approach this equilibrium, with equilibrium levels approximated via linear regression on the lower and upper quantiles of presumed steady-state cells. It is applicable to biological contexts where the transcriptional phase is long enough to approach a steady-state equilibrium. For example, in the process of embryonic stem cell differentiation, when the cells are in a relatively long- term differentiation stage and the transcriptional process has enough time to reach a near-equilibrium state, the scVelo_Dynamical model can be used to analyze the RNA velocity to understand the dynamic changes of cells during the differentiation process.

scVelo_Stochastic: This model is computationally expensive and solves the full splicing kinetic dynamics for each gene, and it can adapt to diverse scenarios like non -stationary populations by avoiding restrictions such as a common splicing rate or the need for sampled steady states. It is very suitable for biological scenarios with high-complexity and non-stationary cell populations. For example, in the study of tumor microenvironments, where there are a large number of heterogeneous cells, and the cell states are constantly changing, the scVelo_Stochastic model can be used to analyze the RNA velocity of different genes in each cell, so as to better understand the dynamic changes of tumor cells and the surrounding microenvironment cells.

UniTVelo is a statistical framework for RNA velocity inference that models the dynamics of spliced and unspliced RNAs via a top-down design using radial basis functions (RBF), enabling flexible transcription activity profiling; it uniquely introduces a unified latent time across the transcriptome to resolve directional discrepancies between genes, with support for both unified-time and independent modes to adapt to diverse biological datasets.

UniTVelo_Unified: This model employs a gene-shared latent time across the transcriptome, aggregating dynamic information from all genes to reinforce temporal cell ordering, and is particularly effective for datasets with genes lacking strong kinetic traits. It is suitable for biological contexts where the kinetic characteristics of genes are not obvious or the data is relatively noisy. For example, in the study of some rare cell types, due to the limited number of

cells and the possible lack of obvious kinetic characteristics of genes, the UniTVelo_Unified model can be used to integrate the information of all genes to analyze the RNA velocity and then infer the cell development trajectory.

UniTVelo_Independent: UniTVelo_Independent: This model assigns gene-specific latent times, allowing greater flexibility for complex datasets with high signal-to-noise ratios, such as those containing cell cycles or sparse cell types. It is suitable for biological scenarios with complex gene expression patterns and high signal-to-noise-ratio data. For example, in the study of cell cycle-related biological processes, different genes may have different expression patterns at different stages of the cell cycle. The UniTVelo_Independent model can assign specific latent times to each gene according to its expression characteristics, so as to more accurately analyze the RNA velocity of genes during the cell cycle.

DeepVelo: This method is a deep learning-based framework that models continuous-time single-cell transcriptome dynamics using neural ordinary differential equations (ODEs), where a variational autoencoder (VAE) learns nonlinear gene regulatory relationships to map gene expression states to RNA velocity, enabling the prediction of future cell states via ODE integration. It can capture complex nonlinear gene interactions, so it is very suitable for biological scenarios that require accurate prediction of cell future states and involve complex nonlinear gene regulatory relationships. For example, in the study of embryonic development, the differentiation process of cells is regulated by a large number of genes, and there are complex nonlinear interactions between these genes. The DeepVelo model can be used to accurately predict the future development direction of cells and help researchers better understand the molecular mechanism of embryonic development.

Important genes identification

To identify genes potentially explaining cell transitions and inferred lineages, we performed differential velocity t-tests to retrieve genes with cluster-specific differential velocity expression—exhibiting significantly higher or lower levels compared to the remaining population ($P<0.05$). The top 100 genes from these differential velocity t-tests for each individual cluster were defined as cluster-specific important genes.
